# Tiny Sea Anemone from the Lower Cambrian of China

**DOI:** 10.1371/journal.pone.0013276

**Published:** 2010-10-13

**Authors:** Jian Han, Shin Kubota, Hiro-omi Uchida, George D. Stanley, Xiaoyong Yao, Degan Shu, Yong Li, Kinya Yasui

**Affiliations:** 1 State Key Laboratory of Continental Dynamics, Department of Geology, Early Life Institute, Northwest University, Xi'an, Shaanxi, People's Republic of China; 2 Seto Marine Biological Laboratory, Field Science Education and Research Center, Kyoto University, Shirahama, Wakayama, Japan; 3 Kushimoto Marine Park Center, Kushimoto, Wakayama, Japan; 4 Department of Geosciences, The University of Montana, Missoula, Montana, United States of America; 5 Key Laboratory of Western China's Mineral Resources and Geological Engineering, School of Earth Science and Land Resources, Chang'an University, Xi'an, Shaanxi, People's Republic of China; 6 Marine Biological Laboratory, Graduate School of Science, Hiroshima University, Higashi-hiroshima, Hiroshima, Japan; Institute of Evolutionary Biology (CSIC-UPF), Spain

## Abstract

**Background:**

Abundant fossils from the Ediacaran and Cambrian showing cnidarian grade grossly suggest that cnidarian diversification occurred earlier than that of other eumetazoans. However, fossils of possible soft-bodied polyps are scanty and modern corals are dated back only to the Middle Triassic, although molecular phylogenetic results support the idea that anthozoans represent the first major branch of the Cnidaria. Because of difficulties in taxonomic assignments owing to imperfect preservation of fossil cnidarian candidates, little is known about forms ancestral to those of living groups.

**Methods and Findings:**

We have analyzed the soft-bodied polypoid microfossils *Eolympia pediculata* gen. et sp. nov. from the lowest Cambrian Kuanchuanpu Formation in southern China by scanning electron microscopy and computer-aided microtomography after isolating fossils from sedimentary rocks by acetic acid maceration. The fossils, about a half mm in body size, are preserved with 18 mesenteries including directives bilaterally arranged, 18 tentacles and a stalk-like pedicle. The pedicle suggests a sexual life cycle, while asexual reproduction by transverse fission also is inferred by circumferential grooves on the body column.

**Conclusions:**

The features found in the present fossils fall within the morphological spectrum of modern Hexacorallia excluding Ceriantharia, and thus *Eolympia pediculata* could be a stem member for this group. The fossils also demonstrate that basic features characterizing modern hexacorallians such as bilateral symmetry and the reproductive system have deep roots in the Early Cambrian.

## Introduction

The Anthozoa is believed to be a sister group of the other cnidarians commonly known as the Medusozoa [1]–[3] and displays bilateral symmetry in morphology [4], [5], which is suggested by gene expression studies to be an ancestral character related to animals prior to the appearance of bilaterian lineages [6], [7]. To understand eumetazoan history, species from Hexacorallia (Zoantharia), the major group of the Anthozoa, have been studied intensively [8]–[10]. However, large ambiguities within hexacorallian phylogeny [11], [12] hinder the evolutionary reconstruction of this group. Shared traits of modern hexacorallians such as mesenterial patterns and cnidae necessary for a coherent phylogenetic understanding are rarely attainable mostly because of the mosaic distribution of the characters [11]. Molecular phylogenetic results based primarily on ribosomal RNA-coding DNA (rDNA) sequences vary and are not much better than those based on comparative morphology [11], [13]–[16].

Fossil records of hexacorallian candidates are relatively abundant because of their skeletal hard parts. Possible hexacorallian orders with calcified skeletons from the Ordovician to Permian such as Rugosa and Tabulata are assumed to have evolved from Cambrian anemone-like ancestors [17] rather than coral-like calcified forms such as the Cothoniida and Tabulaconida [18]. These skeletalized fossils likely represent independent episodes of calcification [19], and all Paleozoic calcified orders of anthozoans became extinct by the end of the Permian, apparently without any direct progeny [20]. The skeleton of coral-like animals is assumed to be ephemeral [21], and thus living corals are likely related to soft-bodied anemone-like forms that go back deeply into the Paleozoic [20]. Soft-bodied fossil records, however, provides only limited insight into phylogenetic relationships because of its rarity and biases of preservation. Ediacaran trace fossils including *Beltanelliformis brunsae*, *Bergaueria sucta* [22]–[24], and recently reported relatively long trail fossils from Newfoundland [25] have been attributed to possible actiniarian trails, but animals that have produced these traces are lacking. Soft-bodied impression fossils from the Lower or Middle Cambrian strata such as *Archisaccophyllia* [26], *Xianguangia* [27] and *Mackenzia* [28] have been attributed to actiniarians based only on their external morphology. Minute possible hexacorallian candidates have been reported even from Ediacaran Weng'an sediments in the Late Neoproterozoic [29]. Most of the globular forms showing some internal structures from Weng'an have later been assumed, however, to be non-biogenetic products [30], [31].

Phosphorite deposits in the lowest Cambrian sediments of the Kuanchuanpu Formation, Shaanxi, China, yielded well preserved soft-bodied microfossils including early metazoan eggs and developing embryos [32], [33]. Their discoveries led to a reconsideration of the nature of the Cambrian diversification [34]. To examine the possibility of soft-bodied microfossil preservation, experiments were conducted on the process of the soft-bodied fossilization. It was found that sulfur oxidizing bacterial interactions in phosphate-enhanced seas in the Early Cambrian could have produced the observed preservation at the cellular level [35]. Furthermore, taphonomic investigations clarified the parameters under which phosphate preservation was possible [36], [37]. We here report soft-bodied sea anemone-like microfossils from the Kuanchuanpu Formation representing the oldest stem hexacorallians directly comparable to extant counterparts.

## Results

### Systematic Paleontology

Phylum Cnidaria Milne-Edwards et Haime, 1857

Class Anthozoa Ehrenberg, 1834

Subclass Hexacorallia Haeckel, 1866

Order and Family incertae sedis

*Eolympia pediculata* Han, Yao, Kubota, Uchida et Yasui gen. et sp. nov.

urn:lsid:zoobank.org:act:F57887A7-FC32-4978-AB5C-769C2ECCF8B6 for *Eolympia*, urn:lsid:zoobank.org:act:3E39708D-6AF1-4A1A-98CD-61F730D44925 for *Eolympia pediculata*.

#### Etymology

Generic name is to commemorate the Olympic games held in Beijing in 2008 when we identified the fossil, which is prefixed by the Greek word ‘eos’ (dawn). The specific name is taken from the remarkable stalk-like pedicle that characterizes the animal.

#### Holotype

Sn27-4 deposited at the Early Life Institute, Northwest University, Xi'an, China.

#### Paratype

Sn52- 58, Sn27- 2, Sn39-1, Sn40-128, Sn27-13, Sn64-83, and Sn76-11 deposited at the same institute as that of the holotype (e-mail: elihanj@nwu.edu.cn).

#### Locality and horizon

Ningqiang, Shaanxi Province, China; *Anabarites trisulcatus*-*Protoherizina anabarica* Zone, Kuanchuanpu Formation, the Fortunian Stage of the Terreneuvian Series (thus the lower unit of the Lower Cambrian).

#### Diagnosis

Animal divided by a circumferential groove into a lower, stalk-like pedicle and an upper, cylindrical body, each body with a whorl of 18 tubercular tentacles on the upper outer margin. The upper body also divided into two cylinders by a weak groove. Internal space partially or completely partitioned by 18 radially arranged mesenteries. Mesenteries frequently fused medially and basally making Y-shaped pairs. Tentacles arranged in alternating position with mesenteries.

### Description

**Figure 1:**
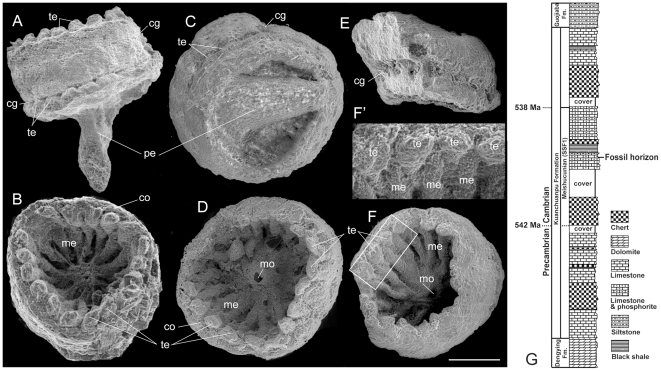
Holotype and paratypes of *Eolympia pediculata*, and stratigraphic scheme. **A**, Lateral and **B**, oral views of the holotype (Sn27-4). **C**, Aboral and **D**, oral views of the stalked paratype (Sn52-58). **E**, Lateral and **F**, oral views of the cylindrical paratype (Sn27-2). **F'**, Magnification of the upper margin (rectangle in **F**) of the cylindrical paratype showing alternate positioning of tentacles and mesenteries. **G**, Stratigraphy [57] and radioisotopic age determination near the upper boundary of the SSF1 (Small Shelly Fossil 1 Zone) [58], which corresponds to *Anabarites trisulcatus* – *Protohertzina anabarica* Assemblage Zone. The Precambrian/Cambrian boundary is tentatively noted. cg, circumferential groove; co, collar; me, mesentery; mo, mouth; pe, pedicle; te, tentacle. Scale bar, 0.2 mm.

**Figure 2:**
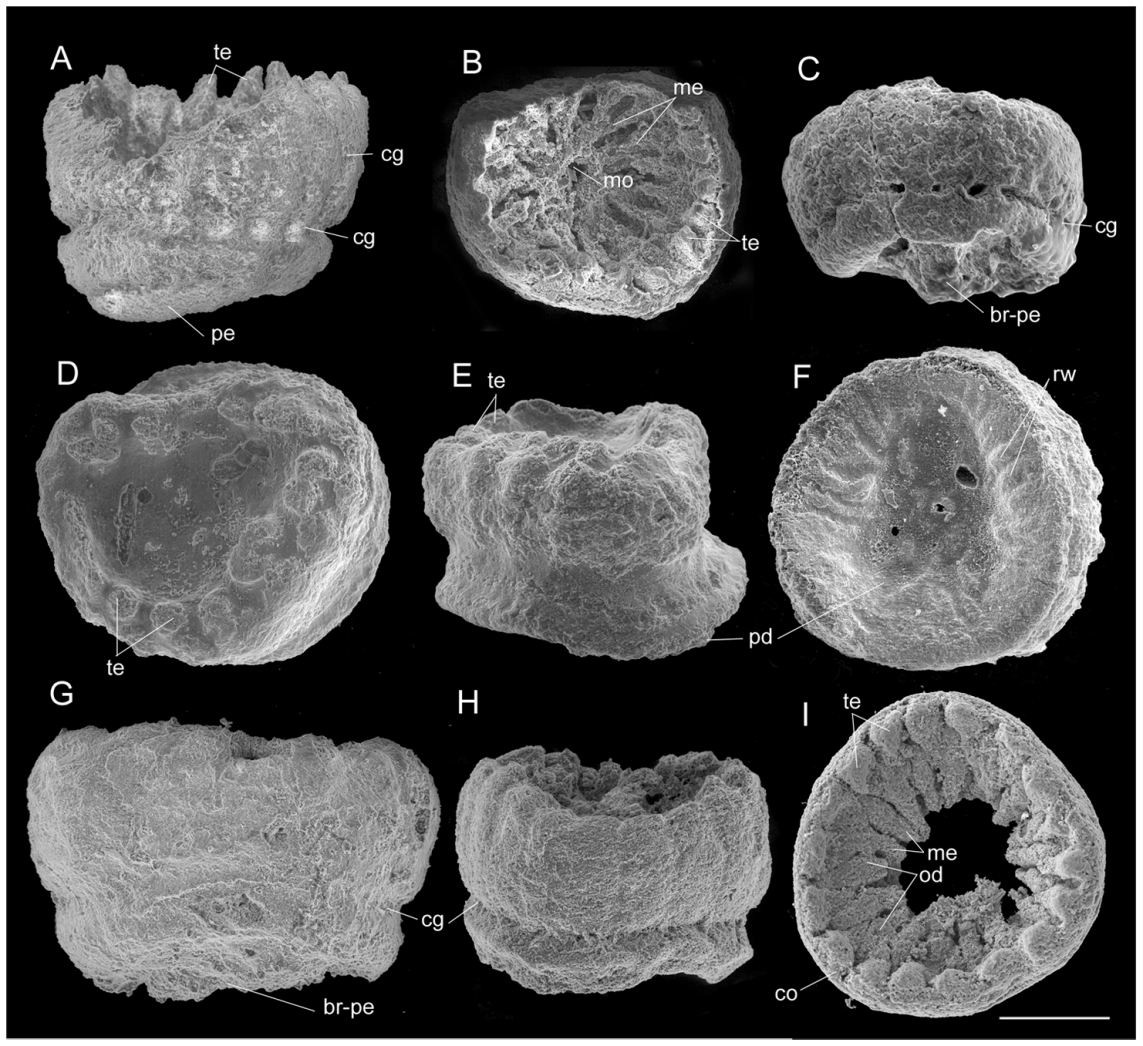
Paratypes of *Eolympia pediculata*. **A**, Lateral view of stalked paratype (Sn52-58). **B**, Oral and **C**, lateral views of stalked paratype (Sn64-83). The stalk is broken at the base. **D**, Oral, **E**, lateral, and **F**, aboral views of reel-shaped paratype (Sn39-1). **G**, Lateral view of compressed paratype (at the largest diameter) that was broken at the pedicle (Sn40-128). **H**, Lateral view of cylindrical paratype (Sn27-13). **I**, Oral view of discoidal paratype preserving partial oral disc and mesenteries (Sn76-11). br-pe, broken pedicle; cg, circumferential groove; co, collar; me, mesenteries; mo, mouth; od, remnant of oral disc; pe, pedicle; pd, pedal disc; rw, radial wrinkle; te, tentacle. Scale bar 0.2 mm.

*Eolympia pediculata* is a minute tentaculated polyp of a solitary form. We present two calycimorph fossils including the holotype, Sn27-4 (Figure 1A, B) and a paratype, Sn52-58 (Figures 1C, D, 2A), two calycimorphs but probably broken at the stalk-like pedicle, Sn64-83 and Sn40-128 (Figure 2B, C, G), three cylindricals, Sn27-2, Sn27-13, and Sn76-11 (Figures 1E, F, 2H, I), and a reel-shaped specimen, Sn39-1 (Figure 2D–F). There are eight specimens in total. The fossils range from 500 to 670 µm in diameter and from 300 to 625 µm in height with or without pedicle. A striking feature is the circumferential grooves on the external body surface, which divide the body into three parts in the stalked specimen and two parts in the cylindrical paratype (Figures 1A, C, E, 2A). At the well-developed groove between the upper body and the lower pedicle in the holotype, 18 tubercles are developed on the upper margin of the lower pedicle, interpretable as tentacles, which are also found in the stalked paratype (Figure 1A, C). Another set of 18 tentacles aligned in a single whorl occur on the upper margin of the upper body. Weak longitudinal reliefs on the external surface of the upper body probably correspond to mesenterial attachments (Figure 1A). On the oral surface, radial ridges extend from the intertentacular space on the column wall toward a central opening, here is interpreted as a mouth (Figures 1B, D, F, F', 2B). The tentacle whorl is surrounded by a collar in some specimens as in modern actiniarians (Figures 1B, D, 2I). The oral disc, which would have been externally flattened or domed in life, has been depressed probably by a postmortem deformation and has come to lie on the underneath mesenteries making their radial relief. Between radial ridges, the oral disc has been invaginated deeply or ruptured exposing internal gastric cavity (Figures 1B, D, F, 2B, I).

**Figure 3:**
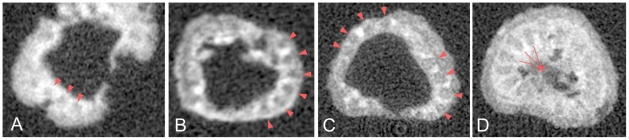
Micro-CT images showing hollow cavities in tentacles. Arrowheads denote hollow cavity that expands fully in tentacles in **A** (Sn40-128), **B** (Sn27-13), and **C** (Sn39-1), and lines with asterisk indicate possible expansion of gastric cavity into tentacles in **D** (Sn39-1).

**Figure 4:**
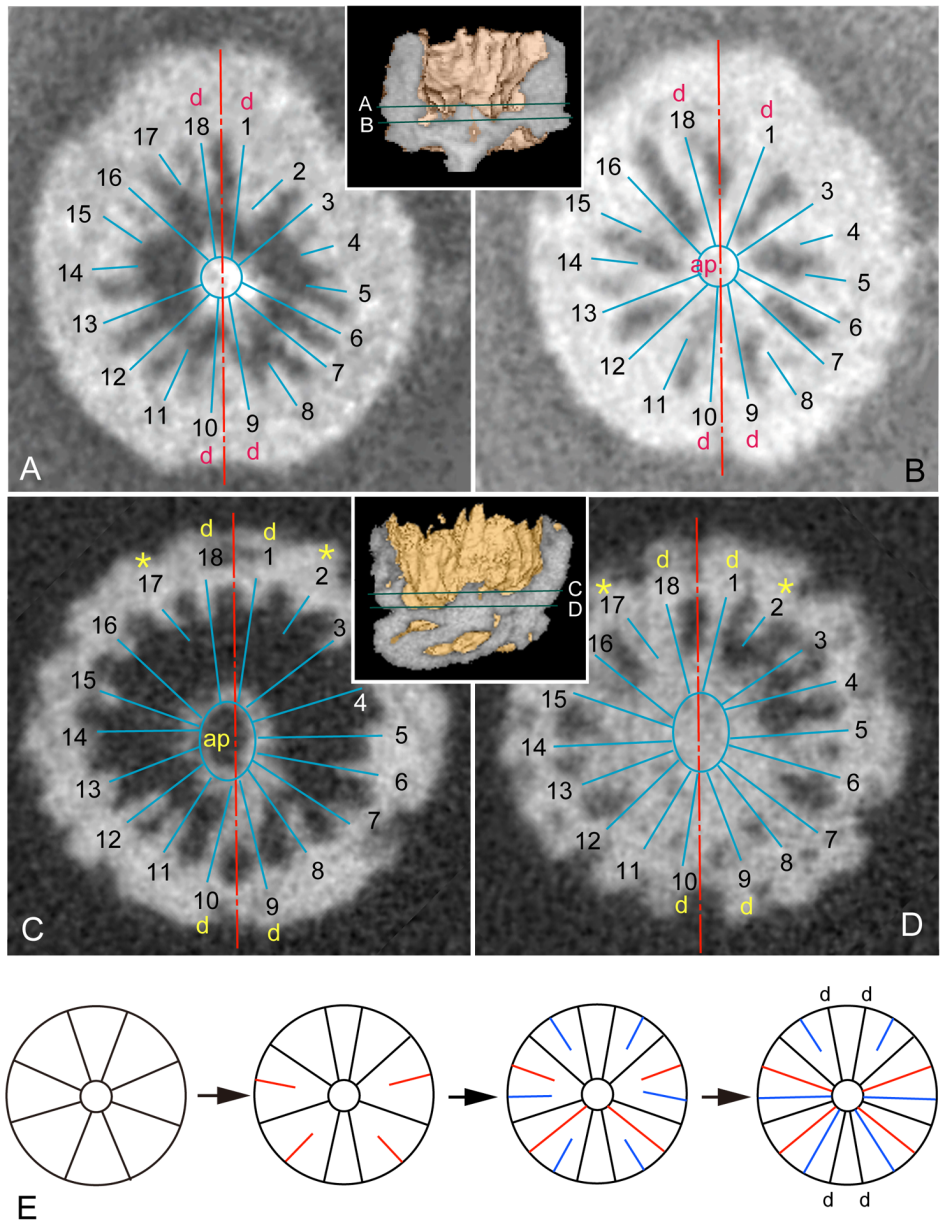
Mesenterial arrangements. **A–B**, Two representative micro-CT transverse sections in the holotype denoted in the upper inset. Ten complete (long blue lines) that fused with the actinopharynx (ap) and eight incomplete (short blue lines) are identified, which are arranged in a bilateral symmetry with axis (red line) through two sets of directives (d). **C–D**, Micro-CT transverse sections in the stalked paratype (Sn52-58) denoted in the lower inset. All but two (Nos. 2 and 17 with asterisks) mesenteries are complete, which can be derived from the holotype pattern. **E**, A developmental pattern supposed from the mesenterial arrangement in the holotype and the paratype. From eight complete mesenteries (black line), four unpaired secondary mesenteries (red line) are developed and acquired hexameral 12 mesenteries. Additional six mesenteries (blue line) appear again in unpaired fashion to complete the system with 18 mesenteries.

Computer-aided microtomographic (micro-CT) analyses allowed us to peer deeper into the polyp. In serial transverse sections in all specimens that were subjected to the micro-CT analysis (Movies S1, S3, S5-7), 18 mesenteries extending from the column wall toward the central mass, which seems to correspond to the actinopharynx, were identified. There are no other internal structures such as calcitic septa as found in corals. This confirms that the radial pattern in the oral view reflects a mesenterial arrangement. The micro-CT image of the reel-shaped specimen has revealed lumens in the tentacles (Figure 3, Movie S6) suggesting hollow tentacles, some of which are traceable into the depressed gastric cavity (Figure 3D). Hollow tentacles were also depicted in the other two paratypes in the micro-CT images (Figure 3A, B). All of the hollow cavities, when identified, occupy full of the tentacles. These observations suggest that the gastric cavity originally extended into the tentacles in the present animals. In the holotype, 10 mesenteries extending to the actinopharynx and eight shorter ones were identified (Figure 4A, B, Movies S1, S2). The former were interpreted as complete mesenteries and the latter incomplete ones, respectively. In the stalked paratype, the central ellipse, undoubtedly regarded as the actinopharynx, has been fused with 16 mesenteries, and leaving only two are incomplete (asterisks in Figure 4C, D, Movies S3, S4). Comparing the mesenterial patterns of the two specimens, we hypothesize that the holotype represents a younger stage than the stalked paratype, with the mesenterial pattern exhibiting a bilateral symmetry with two sets of directives (Figure 4). The number of 18 mesenteries is exceptional, but not out of line for extant hexacorallians. They are found in the Edwardsiidae [38] and the Gonactiniidae [39]. The developmental pattern of the fossil revealed from the CT-images is consistent with that found in Actiniaria+Corallimorpharia+Scleractinia. In these groups, when hexameral pattern of mesenteries (six pairs of complete mesenteries) is established from the stage with four pairs of complete mesenteries, all successive mesenteries appear in a coupled and unpaired manner. After the completion of the hexameral pattern, additional mesenteries appear as coupled pairs [11]. If we apply this plan to *Eolympia pediculata*, 18 mesenteries could not possibly have been formed unless the additional mesenteries appeared after the completion of the hexameral pattern in an unpaired manner as in the young stage of modern Actiniaria+Corallimorpharia+Scleractinia (Figure 4E).

A curious observation in the specimens was that the 18 mesenteries are aligned as in pair and most adjoining mesenteries have fused medially and aborally displaying Y-shaped sections in the micro-CT analysis (Movies S1, 3), which is reflected to the oral views (Figure 1B, D, F). We first considered that this might be resulted from the flexible free end of incomplete mesenteries being attached to an adjoining mesentery; however, constantly observed pair-wise pattern indicates that it is not an artificial character. Extant ptychodactiarian members in the Actiniaria [40] possess the same feature as found in *Eolympia pediculata*, though the number of the mesenteries in the former is 12 or 24, and we thus interpret that the fused mesenteries in *Eolympia pediculata* are original structures attributable to a hexacorallian character.

The stalk-like pedicle is rather slender tapering toward the tip (Figure 1A, C) and extends from the center of the body in the holotype and two broken paratypes (Figures 1A, 2C, G). However, in the most complete paratype (Sn52-58), the stalk has been turned horizontally from the base that is located near the margin of the bottom (Figures 1C, 2A). This eccentric position might be resulted from postmortem deformation and suggests that the body was flexible in life like modern sea anemones. Micro-CT analysis identified a pedicular lumen that opens into the gastric cavity via a narrow canal (Figure 4 lower inset, Movie S4). On the internal surface of the pedicular lumen, there are several longitudinal ridges. There is no attachment disc at the tip of the pedicle.

## Discussion

### Phylogenetic position

**Figure 5:**
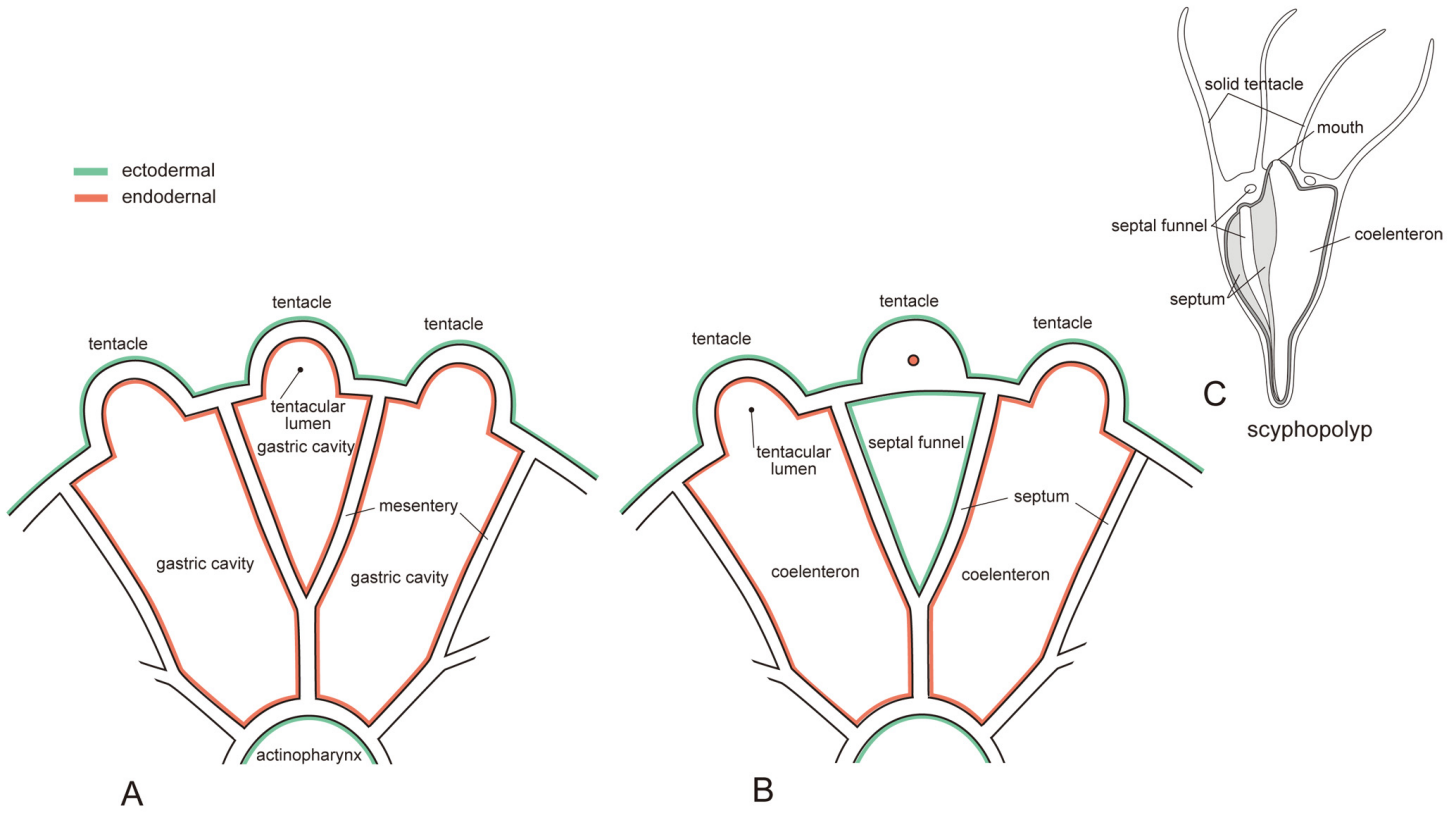
A schematic drawing contrasting two possibilities on spaces between mesenteries (septa). **A**, Adjoining mesenteries are fused medially and aborally making a endodermal pouch continuing to tentacular lumen in hexacorallian case. **B**, Scyphopolyp hypothesis showing an expanded ectodermal septal funnel in a bifurcated septum. A tentacle located at the septal funnel should be solid. **C**, Tetra-radial modern scyphopolyp redrawn from [59].

The oral view of the present fossils and micro-CT images show a deeply depressed oral surface with radial ridges as described above. Between each two fused ridges a deeply invaginated pocket is found frequently. The pocket can be comparable to the septal funnel of typical scyphopolyps [5], though the number of their septal funnels is four without exception. If we accept a phylogenetic relationship between the invaginated pocket in *Eolymipa pediculata* and the septal funnel, 18 mesenteries (septa) create the medial fusion resulting in nine Y-shaped mesenteries (septa), so the space between the two arms from the body column has to be a septal funnel. This idea is, however, denied because the tentacles located in between the two arms of the Y-shaped mesenteries (septa) are also hollow *in Eolympia pediculata*. If septal funnel were the case, tentacles located at septal funnels would have no room to continue their lumen to the gastric cavity (Figure 5). Septa in scyphopolyps have free distal end, which is also different from the configuration of the present fossils. The distal margin of all long mesenteries fused with actinopharynx in the latter. Longitudinally iterated patterns in the present fossils seemingly comparable to strobilation suggest again a scyphozoan affinity. However, we can identify internal structures including hollow that occupies fully the tentacle, mesenteries that have fused medially with an actinopharynx exhibiting two sets of directives and thus bilateral symmetry, and alternate positioning of tentacles and mesenteries. Since the set of these characters never occur in extant medusozoans, the present fossils apparently belong to the Anthozoa.

**Figure 6:**
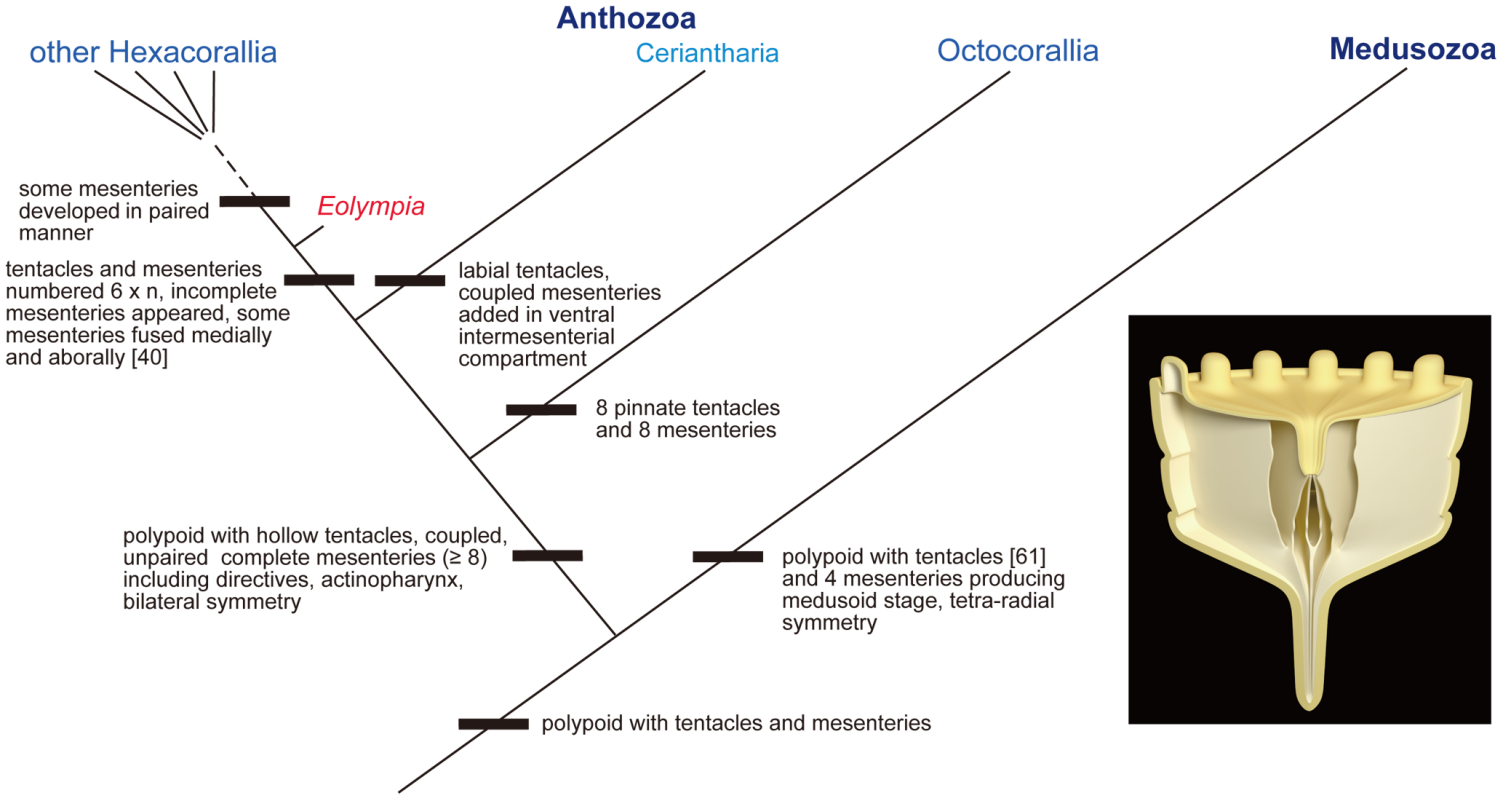
A cladogram showing the position of *Eolympia pediculata* and graphic reconstruction of the animal. Characters noted in the tree are restricted in those related to the present fossils. All diagnostic characters of the present fossils are shared with hexacorallians. The anthozoan and medusozan clades within the Cnidaria are adopted from [60].

The Anthozoa comprises two subclass groups, Hexacorallia and Octocorallia. All of the modern octocorallians have eight pinnate tentacles and the same number of mesenteries. We may thus exclude the possibility of an octocorallian affinity of *Eolympia pediculata*, if we do not hypothesize that these octocoral-specific characters have derived from hexacorallian-like ancestors. Furthermore, there is a recent study on mitochondrial genomes suggesting that the Octocorallia is more closely related to the Medusozoa than to the Anthozoa [3]. The other group Hexacorallia contains six orders. The phylogenetic reconstruction within this group has been in debate because of the lack of any helpful derived characters in these animals [11]. Even in this situation, the Ceriantharia display some specific features such as labial tentacles and continuously forming coupled, unpaired complete mesenteries in the intermesenterial compartment that is located on the opposite (ventral) side of the siphonoglyph. These specific characters suggest that the Ceriantharia may be a sister group of the other hexacorallians [11]. Molecular phylogenetics using rDNA sequences [13]–[15], [41] and comparison of nematocysts [42] also support this phylogeny. On the contrary, all other hexacorallian members and *Eolympia pediculata* develop both complete and incomplete mesenteries, and the number of the tentacles and mesenteries is multiples of six, in general. The only difference between *Eolympia pediculata* and Hexacorallia without Ceriantharia is that mesenteries in the former were formed in an unpaired manner from the hexameral stage onward. Furthermore, we were unable to identify any characters specific to *Eolympia pediculata* other than its minute size. Our phylogenetic interpretation is, therefore, that the present fossil represents an animal that is a stem group of the Hexacorallia, in which the Ceriantharia may have diverged before the appearance of *Eolympia pediculata* (Figure 6).

### Comparison with other fossil hexacorallian candidates

Possible existence of sea anemone-like animals in the Ediacaran has been suggested on the basis of trace fossils [22]–[25]. These trace fossils are cm-size, but no convincing body fossils occur alongside them. Body fossils named *Persimedusites chahgazensis* about one centimeter in diameter from the Ediacaran of Iran have been assigned to the Scyphozoa [43]. They have preserved tentacle-like structures and about 20 radial lobes exhibiting a similar appearance to the oral view of *Eolympia pediculata*. However, there is no lateral impression on this animal, and oral arms identified in the fossils make an actiniarian affinity difficult to accept.

A microfossil from a slightly higher horizon than that of the present fossils at the same locality has been regarded to be a cnidarian polyp without taxonomic assignment [33]. Although the external appearance is similar to our fossils including the number of tentacle-like processes, strobilation-like appearance and the body size, close observations on the SEM images (courtesy by Dr. Steiner) have revealed features distinct from those of *Eolympia pediculata* (Figure S1). Tentacle-like processes in the Steiner and others' fossil can be divided into two types. First are long filiformic tentacles extending from the oral disc. The base of most tentacles of this type show a longitudinal depression that suggests a possible hollow at the base, but the hollow did not extend toward the tip of the tentacle. The other type is found at the upper outer margin of the lower body, which apparently corresponds to the furrow of strobilation. All processes in this type are notched like lappets in modern ephyrae. At the notch, a sense organ rhopalium might be developed later. The polyp found by Steiner et al. [33], based on these features, thus seems to be more likely assigned to scyphozoan primary polyp under strobilation and is distinct from *Eolympia pediculata*.

*Xianguangia sinica* from the Lower Cambrian Chengjiang in China has been reported as a soft-bodied sea anemone [27], [44]. Observation on our new materials attributed to *X. sinica* has identified a holdfast and feather-like tentacles that have many long branches extending alternately from the tentacle axis. These features suggest that *Xianguangia* might be an Ediacaran survivor, a case similar to *Stromatoveris* [45], and thus its affinity to soft-bodied sea anemones [44] is difficult to accept. *Archisaccophyllia* from the Lower Cambrian Chengjiang biota is a possible Cambrian actiniarian anemone with 12 tentacles and longitudinal bands on the body column but no preserved mesenteries [26]. Even though there are no internal structures available, a multiple of six in the number of tentacles in the single whorl remains a possibility of phylogentic relationship between *Archisaccophyllia* and *Eolympia pediculata*.

Fossils that we also need to consider include a variety of Paleozoic corals. They include two major groups, the Tabulata and Rugosa along with minor groups, Lower Cambrian calcified corals informally referred to as coralomorphs [46], and rare Paleozoic scleractiniamorphs including the Ordovician kilbuchophyllids [47] and Permian numidiaphyllids [48]. These Paleozoic animals were believed to have no relation with modern corals and were extinct before the scleractinian radiation [19], [49]. In most Paleozoic corals, six protosepta (secreted hard skeleton being different from mesenteries) are identified like in modern scleractinian corals. However, the Rugosa and Tabulata are distinguished from modern corals in their serial insertion of septa, which resembles modern zoanthinarian pattern, as well as in their calcitic skeleton [49]. On the contrary, the Kilbuchophyllida and Numidiaphyllida show types of cyclic insertion like modern scleractinian corals, but have no features suggesting that they might be direct ancestors of Triassic scleractinians [19], [49]. Although some Paleozoic coral-like forms skeletalized with bifurcated or branched septa [50], these septa have been inferred to be produced by usual paired mesenterial patterns [50] and thus have no relation to the Y-shaped mesentery of *Eolympia pediculata*. Regardless of how these Paleozoic corals are classified within the Anthozoa, all septal patterns display bilateral and six-fold symmetry and suggest some relationships with the present fossils.

### Possible life cycle

The size of the polypoid fossils we describe is minute, but surprising details are present. We have considered if they represent immature forms, but the consistent number of tentacles and mesenteries, as well as the circumferential grooves that preserve ongoing transverse division make an adult interpretation plausible. Strobilation is not restricted to the Scyphozoa. Transverse fission has been reported in some extant hydrozoans and is present in at least four orders of anthozoans [51]. Among living actiniarian species, though longitudinal fission is the primary mode of asexual proliferation [52], the tiny species *Gonactinia prolifera* displays a chain of clonal individuals, each discernible by its whorl of tentacles before transverse fission [53]. This is comparable to the strobilation we have observed in *Eolympia pediculata*.

**Figure 7:**
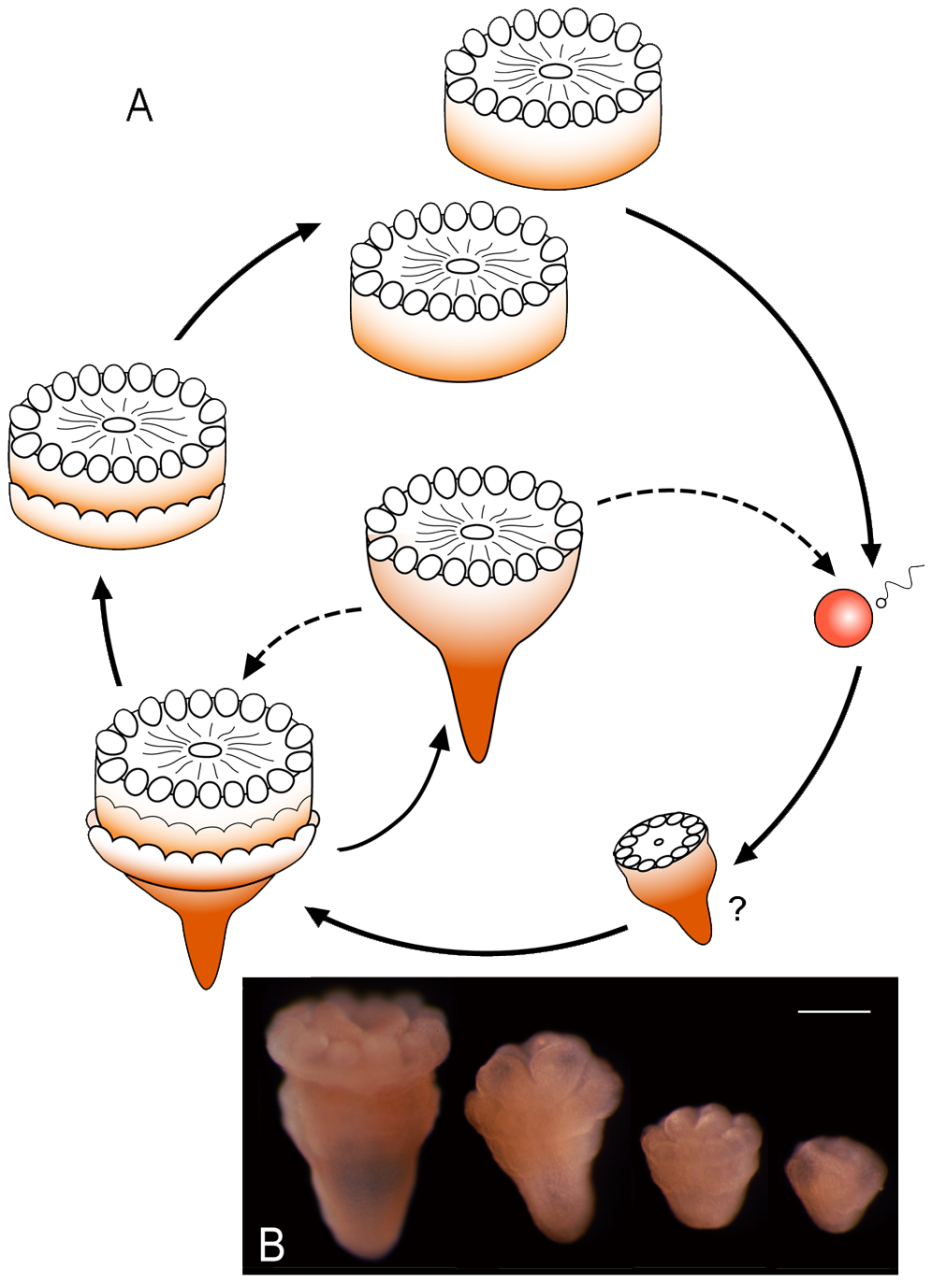
A schematic drawing suggesting the life history and young polyps of an extant viviparous species. **A**, The fossils demonstrate asexual proliferation by two-round transverse fission. Transverse fission may occur in the juvenile and adult stage. The stalk-like pedicle of the holotype and a paratype also implies sexual life cycle of the animal. **B**, Young polyps obtained from the gastric cavity of an extant actinostolid actiniarian species from 192–250 m in depth. Scale bar, 0.2 mm.

Because the cylindrical bodies possess a circumferential groove before and after the separation from the pedicle body, it suggests that clonal proliferation in *Eolympia pediculata* occurred first as a doublet and then divided into two single individuals (Figure 7A). The reel-shaped paratype (Figure 2D–F, Movie S6) represents a stage after the final division, which shows a definitive pedal disc with radiating wrinkles. The stalk-like pedicle of the present fossil is intriguing when considering the life history. It is comparable to that of primary polyps immediately after metamorphosis from sexually produced planulae of various cnidarians, as well as of young polyps brooded in the gastric cavity of an undescribed tiny actiniarian species of the Actinostolidae from Japan (Figure 7B). This resemblance suggests that the stalked individuals were reproduced sexually. The present fossils provide evidence that both sexually and asexually reproducing cnidarians had appeared prior to the Cambrian diversification.

### Deep history of anthozoan bilaterality

The cnidarian body plan is often characterized as being radially symmetrical despite many examples of bilaterality, including fossil examples [4], [5], [54]. However, gene expression studies have clarified that many genes asymmetrically expressed in deuterostomes and protostomes are commonly utilized in the developing actiniarian *Nematostella vectensis*, suggesting that the axial characteristics of this species could represent that of the common ancestor of cnidarians and bilaterians [7]. Interestingly, asymmetric gene expression in *N. vectensis* coincides with the directive axis, an axis through the two sets of directives. This suggests that the directive axis is homologous with the dorso-ventral axis of bilaterians [7], [55]. The bilateral symmetry of the mesenterial pattern recognized by the directives in *Eolympia pediculata* provides evidence supporting the speculation derived from the gene expression studies [7], [55]. Bilateral symmetry characterized by two pairs of directives must have been acquired in a critical time early in the radiation of cnidarians, and this likely led to the mesenterial and septal patterns observed in subsequent fossil groups and also in present-day hexacorallians produced by the serial and cyclic insertion of paired or unpaired mesenteries [17].

Although the present fossils are the oldest that are directly comparable to modern hexacorallians, the polypoid fossil from the same locality [33] that might be at scyphozoan grade, and medusa fossils from the Middle Cambrian in Utah exhibiting characters comparable to modern jellyfish taxa [56] have also been found. The cnidarian diversification might have occurred rather quickly during the early half of the Cambrian or it may be deeply rooted into the Neoproterozoic.

## Materials and Methods

### Rock treatments and observations

The rock samples were collected from the Kua 115–118 [33] in the Kuanchuanpu Formation of the Shizhonggou Section at Ningqiang, southwestern Shaanxi, China, from 2005 to 2008. Collected rocks were treated with 10% acetic acid for three days. Remaining microfossils and other granules were washed with tap water, dried and then sorted under the dissection microscope. Selected microfossils were subjected to scanning electron microscopy. Internal structures were analyzed with micro-CT at about 5-µm resolution power (TXS225-ACTIS, TESCO at University Museum, The University of Tokyo, Japan).

### Nomenclatural acts

The electronic version of this document does not represent a published work according to the International Code of Zoological Nomenclature (ICZN), and hence the nomenclatural acts contained in the electronic version are not available under that Code from the electronic edition. Therefore, a separate edition of this document was produced by a method that assures numerous identical and durable copies, and those copies were simultaneously obtainable from the publication date noted on the first page of this article for the purpose of providing a public and permanent scientific record, in accordance with Article 8.1 of the Code. The separate print-only edition is available on request from PLoS by sending a request to PLoS ONE, 1160 Battery Street Suite 100, San Francisco, CA 94111, USA along with a check for $10 (to cover printing and postage) payable to “Public Library of Science”. Digital archives where the present paper is deposited are PubMedCentral, LOCKSS, Hiroshima University Institutional Repository, and Northwest University Repository. In addition, this published work and the nomenclatural acts it contains have been registered in ZooBank, the proposed online registration system for the ICZN. The ZooBank LSIDs (Life Science Identifiers) can be resolved and the associated information viewed through any standard web browser by appending the LSID to the prefix “http://zoobank.org/”. The LSID for this publication is: urn:lsid:zoobank.org:pub:E78F2669-6D58-4B0E-84F2-94253ABAF0CF.

## Supporting Information

Figure S1 Polypoid fossil (Kua125-56) of Steiner et al. and its affinity. The fossil [33] from an upper horizon at the same locality as that of the present fossils displays a scyphozoan affinity represented by filiformic tentacles with proximal hollow of primary polyp, which is suggested by longitudinal groove (white arrowheads), and notched lappet-like processes (pink arrowheads) during strobilation. Scale bar, 0.2 mm.
(10.10 MB TIF)

Movie S1 Serial transverse micro-CT sections of holotype Sn27-4 (QuickTime; 2.1 MB).
(2.17 MB MOV)

Movie S2 Serial sagittal micro-CT sections of holotype Sn27-4 (QuickTime; 2.2 MB).
(2.35 MB MOV)

Movie S3 Serial transverse micro-CT sections of paratype Sn52-58 (QuickTime; 1.9 MB).
(1.97 MB MOV)

Movie S4 Serial sagittal micro-CT sections of paratype Sn52-58 (QuickTime; 2.0 MB).
(2.05 MB MOV)

Movie S5 Serial transverse micro-CT sections of paratype Sn27-2 (QuickTime; 1.4 MB).
(1.43 MB MOV)

Movie S6 Serial transverse micro-CT sections of paratype Sn39-1 (QuickTime; 1.9 MB).
(1.39 MB MOV)

Movie S7 Serial transverse micro-CT sections of paratype Sn27-13 (QuickTime; 0.73 MB).
(0.75 MB MOV)
