# Supplementary figures and images for: Tiny Sea Anemone from the Lower Cambrian of China
Source: PLoS One. 2010 Oct 13;5(10):e13276. doi: 10.1371/journal.pone.0013276 (PMC2954142; doi:10.1371/journal.pone.0013276)

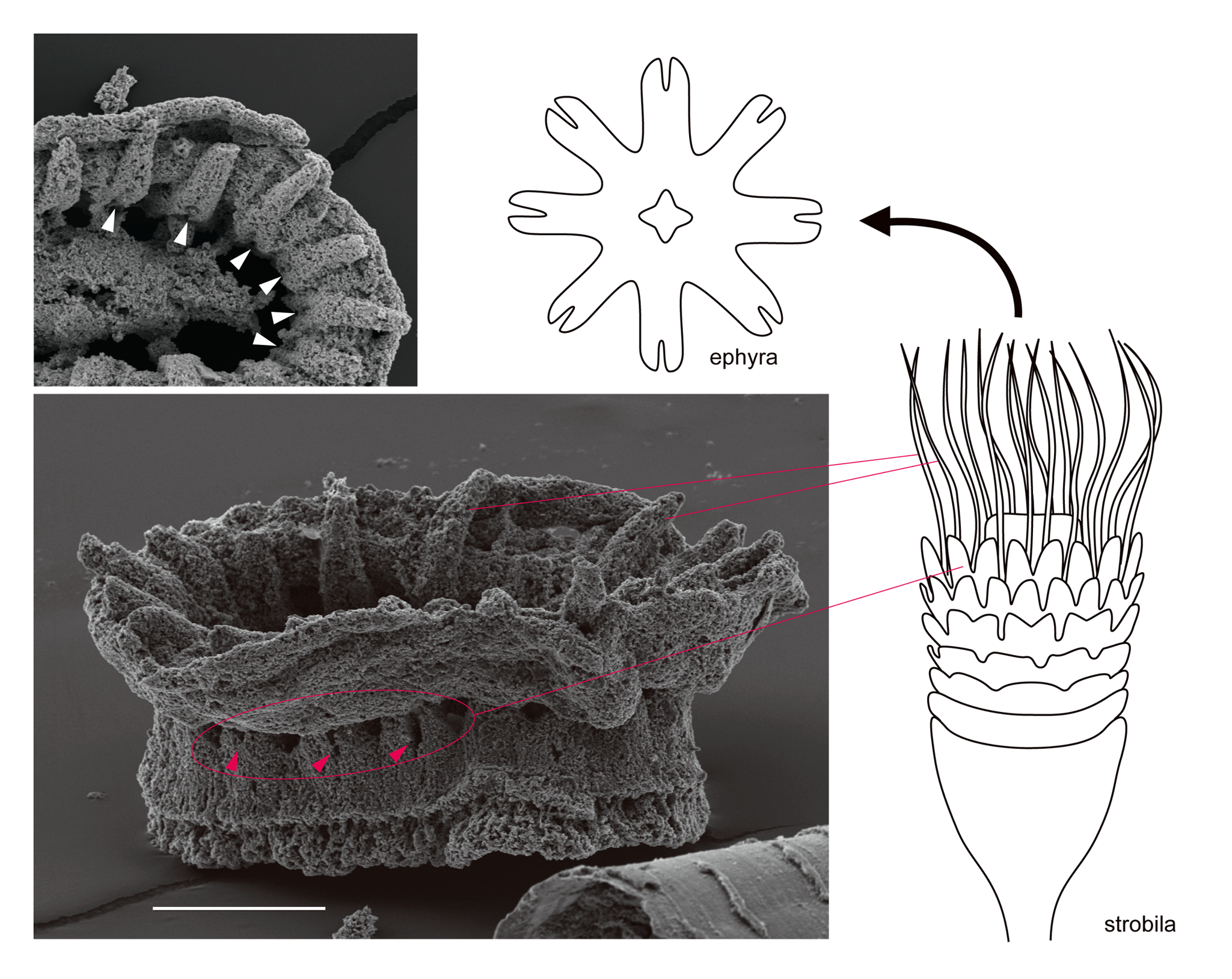

Supplement: Figure S1 — Polypoid fossil (Kua125-56) of Steiner et al. and its affinity. The fossil [33] from an upper horizon at the same locality as that of the present fossils displays a scyphozoan affinity represented by filiformic tentacles with proximal hollow of primary polyp, which is suggested by longitudinal groove (white arrowheads), and notched lappet-like processes (pink arrowheads) during strobilation. Scale bar, 0.2 mm. (10.10 MB TIF) [file pone.0013276.s001.tif]
